# Trends in Public Awareness and Knowledge of Drinking Guidelines: a Representative Population Survey in England, 2016–2022

**DOI:** 10.1093/alcalc/agad007

**Published:** 2023-02-08

**Authors:** Sarah E Jackson, Xiaotang Guo, John Holmes, Jamie Brown

**Affiliations:** Department of Behavioural Science and Health, University College London, London WC1E 6BT, UK; SPECTRUM Consortium, UK; Department of Behavioural Science and Health, University College London, London WC1E 6BT, UK; School of Health and Related Research, University of Sheffield, Sheffield S1 4DE, UK; Department of Behavioural Science and Health, University College London, London WC1E 6BT, UK; SPECTRUM Consortium, UK

## Abstract

**Aim:**

To examine trends in public awareness and knowledge of drinking guidelines in the UK since their revision in 2016, which had moved from a daily to a weekly guideline, made the guideline the same for men and women, and reduced the guideline for men by around one-third.

**Method:**

Data were from a representative, repeat cross-sectional survey. We analysed changes in awareness and knowledge of drinking guidelines among 8168 adult drinkers between 2016 and 2022 and associations with sociodemographic characteristics, smoking status and level of alcohol consumption.

**Results:**

The proportion of drinkers aware of guidelines declined from 86.0% (95%CI 84.0–88.0%) in 2016 to 81.7% (79.5–84.0%) in 2019, then increased during the COVID-19 pandemic, peaking at 91.6% (90.1–93.1%) in 2020. The proportion who correctly identified the guideline as a maximum of exactly 14 units/week remained at around a quarter from 2016 (25.0%, 22.4–27.5%) to 2018 (25.8%, 23.2–28.3%), whereas the proportion who gave a figure of 14 units or fewer rose from 52.1 (49.2–55.0%) to 57.4% (54.6–60.3%). However, by 2022, guideline knowledge had worsened significantly, with these figures falling to 19.7 (17.4–21.9%) and 46.5% (43.6–49.4%), respectively. Changes over time were similar across subgroups. Odds of guideline awareness and knowledge were higher among drinkers who were aged ≥35, female, more educated and from more advantaged social grades.

**Conclusions:**

The majority of adult drinkers in the UK are aware of low-risk drinking guidelines. However, 6 years since their announcement, knowledge of the revised drinking guidelines remains poor. Less than a quarter know the recommended weekly limit and only around half think it is 14 units or less. Inequalities have persisted over time, such that disadvantaged groups remain less likely to know the guidelines.

## INTRODUCTION

Alcohol use ranks among the leading risk factors for death and disability-adjusted life years globally (Murray *et al.*, 2020). As such, drinking at lower levels can substantially reduce the risks to health (Griswold *et al.*, 2018). Health authorities in most high-income countries have issued guidelines on ‘low-risk’ drinking that define levels or patterns of alcohol use, which are associated with a low risk of alcohol-related problems for most healthy adults (Holmes *et al.*, 2019). These guidelines are usually intended to help drinkers to make informed choices about their alcohol use. However, their effectiveness in reducing alcohol harm depends in part on people (i) being aware that they exist and (ii) having knowledge of their recommendations.

In January 2016, the UK’s Chief Medical Officers revised their guidelines for low-risk alcohol consumption (Department of Health, 2016) following a review that formed a central component of the Government’s 2012 alcohol strategy (HM Government, 2012). The new guidelines recommend that men and women should not drink more than 14 units of alcohol per week on a regular basis (1 UK unit = 10 ml/7.9 g ethanol), that those drinking 14 units a week should spread this over at least 3 days a week, and that people should aim to have at least two drink-free days each week (Department of Health, 2016). The previous guidelines, published in 1995, recommended that men should not regularly drink more than 3–4 units of alcohol a day and women not more than 2–3 units a day. Thus, the revision constituted three key changes: (i) moving from a daily to a weekly guideline, (ii) making the guideline the same for men and women, and (iii) reducing the guideline for men by around one-third (Holmes *et al.*, 2020c).

The revised drinking guidelines received limited publicity. They were announced in January 2016 via a Government press release titled ‘New alcohol guidelines show increased risk of cancer’, which emphasized the link between consuming even small amounts of alcohol and cancer risk (Department of Health and Social Care, 2016). This attracted news and social media attention over several months (Holmes *et al.*, 2020a; Kersbergen *et al.*, 2022)—news media generally reported on the guidelines in a neutral and accurate manner, but in-depth coverage was often negative and sought to discredit the guidelines (Kersbergen *et al.*, 2022). Since this initial media campaign, the promotion of the guidelines in the UK has been limited to one brief campaign. In September 2018, Drinkaware launched a campaign promoting the recommendation to have at least two drink-free days per week. This was a UK-wide campaign that was run in collaboration with Public Health England (attracting criticism, given Drinkaware’s strong ties with the alcohol industry; Smyth, 2018). Several years since the new guidelines were announced, many alcohol producers have not updated the guidelines on drink labels: in 2019, more than 70% of labels did not display up-to-date low-risk drinking guidelines and almost a quarter contained misleading, out of date information (Alcohol Health Alliance UK, 2020). A more recent report estimated that only 35% lacked up-to-date labelling, but appeared to use an unrepresentative sample of products (Alcohol Health Alliance UK, 2022).

An early evaluation of short-term trends in awareness and knowledge of drinking guidelines using nationally representative data (Holmes *et al.*, 2016) indicated there was little change in public awareness of guidelines up to May 2016, although awareness was already high at 87% in December 2015. The data showed a significant increase in knowledge of the guidelines as 14 units per week or fewer among men (for whom the weekly guideline changed)—from 23% in December 2015 to 43% in January 2016, falling to 36% by May 2016—but no significant change in knowledge among women (for whom the weekly guideline remained the same) (Holmes *et al.*, 2016). A further analysis of trends through October 2017 similarly showed no substantial or sustained changes in drinkers’ awareness or knowledge of drinking guidelines (Holmes *et al.*, 2020b, 2020c). The primary aim of this study was to update these analyses to examine whether and, if so, to what extent there have been long-term changes in public awareness and knowledge of drinking guidelines since the announcement of the revised guidelines in January 2016.

When examining trends in awareness and knowledge of drinking guidelines, it is important to take into account people’s sociodemographic characteristics and drinking status and evaluate the extent to which changes are experienced equally across the population. Previous studies have indicated differences by gender, education, smoking status and level of alcohol consumption (Office for National Statistics, 2010; Bowden *et al.*, 2014; Buykx *et al.*, 2018 ; Islam *et al.*, 2019). Women, those with higher levels of education and higher alcohol consumption are more likely to be aware of guidelines and less likely to underestimate the recommended unit limit (Buykx *et al.*, 2018; Islam *et al.*, 2019). Smokers are more likely than non-smokers to overestimate the recommended unit limit (Buykx *et al.*, 2018). If certain subgroups have lower levels of awareness or knowledge of drinking guidelines (or smaller improvements over time), this could contribute to health inequalities by increasing their vulnerability to alcohol-related harm. Thus, our secondary aim was to investigate whether sociodemographic characteristics and drinking status moderated long-term trends in awareness and knowledge of drinking guidelines.

Using data from the Alcohol Toolkit Study, we addressed the following research questions:

1. Among adult drinkers in the UK, to what extent have awareness and knowledge of drinking guidelines changed between 2016 and 2022?
2. Has the extent of any changes in awareness and knowledge of drinking guidelines between 2016 and 2022 differed by age, gender, education, occupational social grade, smoking status, or level of alcohol consumption?

## METHOD

### Design and study population

Data were drawn from the ongoing Alcohol Toolkit Study, a monthly repeat cross-sectional survey of a representative sample of adults in the UK designed to provide insights into population-wide influences on drinking behaviour (Beard *et al.*, 2015). The study uses a combination of random location and quota sampling to select a new sample of approximately 1700 adults each month.

Data are usually collected monthly through face-to-face computer-assisted interviews. However, social distancing restrictions under the COVID-19 pandemic meant that no data were collected in March 2020, and data from April 2020 onwards were collected via telephone, and the lower age bound for participation was increased from 16 to 18 years due to changes in consenting procedures. The telephone-based data collection relied upon the same combination of random location and quota sampling, and weighting approach as the face-to-face interviews and comparisons of the two data collection modalities indicate good comparability (Jackson *et al.*, 2021, 2022; Kock *et al.*, 2022).

The Alcohol Toolkit Study collected data on awareness and knowledge of drinking guidelines in selected months between 2015 and 2022 (each wave from November 2015 to October 2017, then reduced to biennial [April 2018, October 2018, April 2019, October 2019] and then annual [April 2020, April 2021, April 2022] assessments thereafter due to funding changes). We restricted our sample to adults (≥18 years) who reported having drunk alcohol in the past 6 months. Our primary analysis used annual data collected in April of each year from 2016 to 2022, to limit seasonal influence on trends over time. This provided a sample of 8168 participants. We also report descriptive data from 2115 participants surveyed in November/December 2015 (combined) to provide an indication of change from before to after the change in guidelines, taking the total sample size to 10,283. Secondary analyses used data from all available waves, providing a sample size of 34,265.

### Measures

#### Explanatory variable

The explanatory variable was survey year, which ranged from 2016 to 2022, and was analysed as a categorical variable to allow for non-linear associations with our outcomes.

#### Outcome variables

Outcome variables were awareness and knowledge of drinking guidelines. Awareness of drinking guidelines was assessed among participants who reported drinking any alcohol in the past 6 months with the question: ‘*As you may be aware, some drinks contain more alcohol than others. The amount of alcohol in a drink is measured in units. Before today, have you ever heard of there being a recommended maximum number of alcohol units people should drink in a day or a week? This is sometimes known as a “drinking guideline”.’* Response options were *yes*, *no* and *don’t know*. We dichotomized responses to distinguish between those who were aware of the guidelines and those who were not aware or responded *don’t know*.

Knowledge of drinking guidelines was assessed among participants who indicated being aware of drinking guidelines before the interview. They were asked about the guideline for their own gender, with the question: ‘*Can you tell me how many units per day or per week that drinking guideline is for men/women?*’ Participants could respond in units per day or per week. We analysed responses in five categories: (i) fewer than 14 units per week (or 2 units per day), (ii) exactly 14 units, (iii) more than 14 units, (iv) aware of but did not know the guidelines and (v) not aware of the guidelines. For some analyses (as described below), we dichotomized responses to distinguish between those who knew the guidelines were equal to or fewer than 14 units per week and those who did not (i.e. those who believed it to be more, did not know, or were not aware of the guidelines).

#### Moderators

Potential moderating variables included age, gender, education, occupational social grade, smoking status and level of alcohol consumption. Age was categorized as 18–34, 35–64 and ≥ 65 years. Gender was self-identified as *male*, *female*, or *in another way*. Data are not reported separately for the latter group because very small numbers meant we were not able to derive precise estimates of prevalence. Education was categorized as any vs. no post-16 qualifications. Social grade was categorized as ABC1 (which includes managerial, professional and intermediate occupations) vs. C2DE (which includes small employers and own-account workers, lower supervisory and technical occupations, and semi-routine and routine occupations, never workers and long-term unemployed). This occupational measure of social grade is a valid index of SES, widely used in research in UK populations (National Readership Survey, 2007; Beard *et al.*, 2019). Smoking status was categorized as current smoker (including daily or non-daily smoking) vs. non-smoker. The level of alcohol consumption was measured using the first three questions of the Alcohol Use Disorders Identification Test (AUDIT-C) (Babor *et al.*, 2001), which measure and score the quantity and frequency of alcohol consumption and frequency of heavy episodic drinking. When summed across all three questions, scores range from 0 (non-drinker) to 12 (heaviest consumption); we included in our sample anyone with a score ≥ 1 (indicating drinking monthly or less during the past 6 months). For descriptive analyses, alcohol consumption was categorized as low risk (score of 1–4) or increasing/higher risk (score ≥ 5).

### Statistical analysis

Analyses were done in SPSS v.27 and R Studio v.1.4.1717. The analyses were not pre-registered and should be considered exploratory. Data were weighted (in all analyses) to match the English population profile on age, social grade, region, tenure, ethnicity and working status within sex. The dimensions are derived monthly from a combination of the English 2011 census, Office for National Statistics mid-year estimates and an annual random probability survey conducted for the National Readership Survey. Missing cases were excluded on a per-analysis basis. We applied a false discovery rate correction (Benjamini and Hochberg, 1995) to all *P*-values to account for multiple testing using an online calculator (https://www.sdmproject.com/utilities/?show=FDR).

We used descriptive statistics to estimate the prevalence and 95% confidence interval (CI) of (i) awareness and (ii) knowledge of drinking guidelines (using the five-category variable), in relation to survey year. We used multivariable logistic regression to analyse associations of survey year, sociodemographic characteristics and drinking and smoking status with odds of awareness and knowledge (using the two-category variable) of drinking guidelines. Then, to explore whether changes in awareness and knowledge of drinking guidelines over time were moderated by sociodemographic characteristics, drinking status and smoking status, we ran a series of logistic regression models in which two-way interactions between survey year and (i) age (18–34 vs. 35–64 vs. ≥65), (ii) gender (male vs. female), (iii) education (any vs. no post-16 qualifications), (iv) occupational social grade (ABC1 vs. C2DE), (v) smoking status (current smoker vs. non-smoker) and (vi) level of alcohol consumption (continuous AUDIT-C score) were added. Our primary analyses focused on data collected in April of each year, to remove any seasonal influence on trends. We also present descriptive statistics on trends using data from all available waves as secondary analyses in Supplementary Material. As a sensitivity analysis, we reran tests of interactions using data from 2016 to 2019 only to remove any influence of the change in modality of data collection in 2020.

## RESULTS

Supplementary Table 1 summarizes sample characteristics in relation to survey year. Overall, slightly over half of drinkers were male, half were aged 35–64 years and the majority had post-16 educational qualifications, were from social grades ABC1, and were non-smokers and low-risk drinkers. The sample profile was fairly consistent across years, with the exception of 2020 when there was a notable increase in the proportion of adults who reported drinking alcohol in the past 6 months (from 68.1% in 2019 to 80.2% in 2020). There was also a short-term shift in the profile of drinkers around this time, which covers the first wave of COVID-19 lockdown restrictions in the UK, with an increase in the proportion who were increasing/higher risk drinkers (34.2, 47.3 and 36.3% in 2019, 2020 and 2021), and a decrease in the proportion who had no post-16 qualifications (38.0, 33.4 and 38.5%, respectively).

**Fig. 1:**
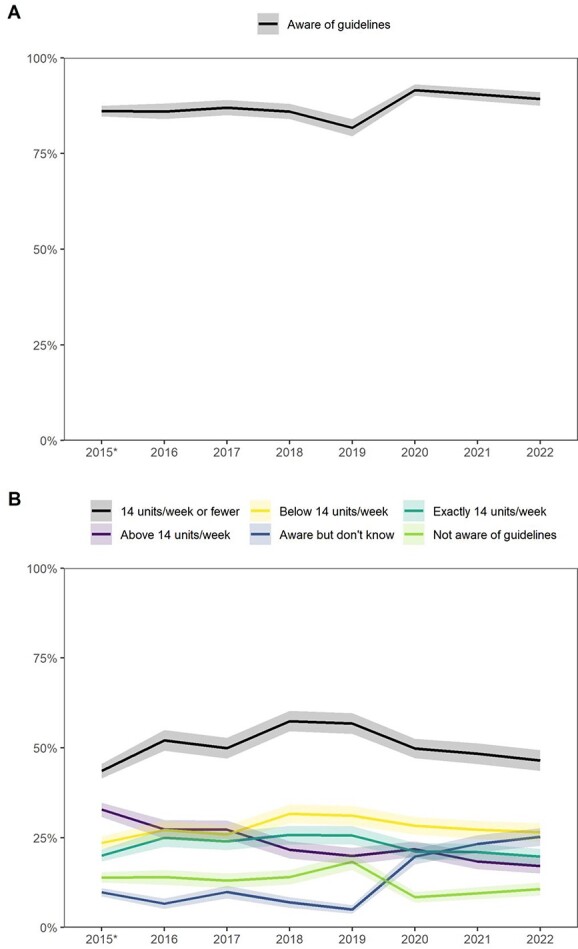
Trends in awareness and knowledge of drinking guidelines among adult drinkers in the UK by survey year. Lines represent the weighted prevalence of (**A**) awareness and (**B**) knowledge of drinking guidelines. Shaded bands indicate the 95% CI. *2015 data are from November and December combined: the only months in which awareness and knowledge of drinking guidelines were assessed. Note: In (B), the line for *14 units/week or fewer* combines respondents who report the guideline to be exactly or below 14 units per week.

Figure 1 shows trends in the prevalence of awareness and knowledge of drinking guidelines by survey year. Percentages and 95% CI are also provided in Supplementary Table 2. In 2016, 86.0% [95% CI 84.0–88.0%] of drinkers said they were aware of drinking guidelines: an unchanged proportion compared with November/December 2015 before the revised guidelines were announced (Fig. 1A). This percentage was stable between 2016 and 2018, before decreasing in 2019, to 81.7% [79.5–84.0%], and then increasing significantly between 2019 and 2020, peaking at 91.6% [90.1–93.1%], before returning to a slight (non-significant) declining trend.

In 2016, just 25.0% [22.4–27.5%] of drinkers were able to correctly identify the guideline as a maximum of exactly 14 units per week and 52.1% [49.2–55.0%] gave a figure of 14 units or fewer (Fig. 1B). Although these proportions were relatively low, they represented a significant change in knowledge relative to November/December 2015, before the revised guidelines were announced (when they were 20.0 [18.4–21.7%] and 43.6% [41.5–45.6%], respectively). After 2016, knowledge of the guideline improved, peaking at 25.8 [23.2–28.3%] and 57.4% [54.6–60.3%], respectively, in 2018. However, by 2022, knowledge of the guideline had worsened significantly, with these figures falling to 19.7 [17.4–21.9%] and 46.5% [43.6–49.4%], respectively. The proportion of drinkers who believed the drinking guideline was *above* 14 units per week also declined significantly over time, from 27.3% [24.7–29.9%] in 2016 to 17.1% [15.0–19.3%] in 2022. There was a substantial increase in the proportion of drinkers who were aware of drinking guidelines but did not know the guideline from 2020 onwards, increasing from 6.6% [5.2–8.1%] in 2016 to 25.2% [22.7–27.7%] in 2022.

Trends in the prevalence of awareness and knowledge of drinking guidelines by survey year followed a similar pattern when data from all available waves were included (Supplementary Fig. 1, Supplementary Table 4).

**Fig. 2:**
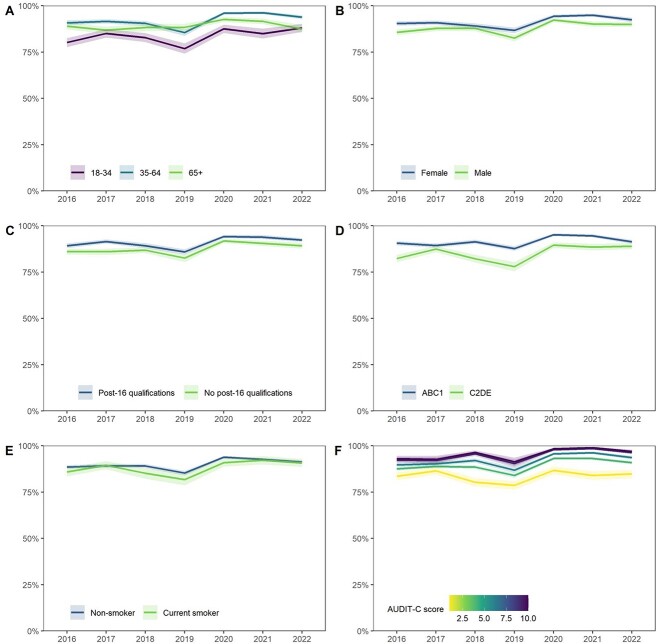
Trends in awareness of drinking guidelines within sociodemographic groups. Awareness is operationalized as the weighted percentage of adult drinkers who have heard of drinking guidelines. Lines represent point estimates from logistic regression allowing an interaction between survey year and (**A**) age, (**B**) sex, (**C**) education, (**D**) occupational social grade, (**E**) smoking status and (**F**) level of alcohol consumption, adjusting for all other variables. Shaded bands indicate the 95% CI.

**Fig. 3:**
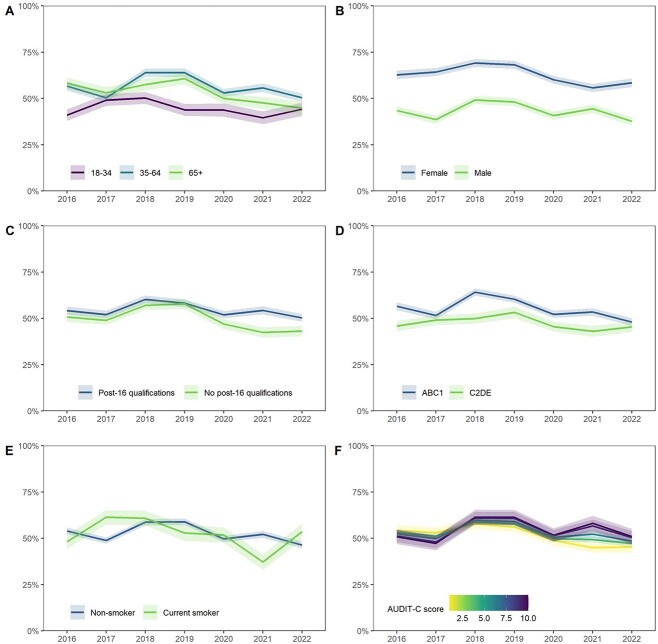
Trends in knowledge of drinking guidelines within sociodemographic groups. Knowledge is operationalized as the weighted percentage of adult drinkers who report the drinking guideline to be 14 units per week or fewer. Lines represent point estimates from logistic regression allowing an interaction between survey year and (A) age, (B) sex, (C) education, (D) occupational social grade, (E) smoking status and (F) level of alcohol consumption, adjusting for all other variables. Shaded bands indicate the 95% CI.

Logistic regression models testing associations of survey year and sociodemographic characteristics with awareness and knowledge of drinking guidelines are shown in Tables 1 (awareness) and 2 (knowledge). Trends in awareness and knowledge of drinking guidelines within sociodemographic groups are shown in Figs. 2 and 3, respectively, and interaction results in Supplementary Tables 5 and 6.

**Table 1:** Odds of being aware of drinking guidelines by survey year, sociodemographic characteristics and drinking status

		**OR [95% CI]**	***P***	**OR**_**adj**_ **[95% CI]**	***P***
Survey year [ref 2016]					
2017		1.12 [0.88–1.43]	0.573	1.15 [0.89–1.48]	0.492
2018		0.99 [0.78–1.25]	0.976	1.07 [0.84–1.37]	0.758
2019		0.73 [0.58–0.91]	0.025	0.77 [0.60–0.97]	0.080
2020		1.95 [1.49–2.55]	<0.001	1.91 [1.45–2.52]	<0.001
2021		1.58 [1.21–2.05]	0.005	1.73 [1.31–2.27]	<0.001
2022		1.40 [1.08–1.82]	0.040	1.42 [1.09–1.86]	0.038
Age (years) [ref 18–34]					
35–64		2.28 [1.95–2.66]	<0.001	2.27 [1.93–2.67]	<0.001
≥65		1.36 [1.14–1.62]	0.005	1.63 [1.35–1.98]	<0.001
Gender [ref male]					
Female		1.32 [1.16–1.52]	<0.001	1.49 [1.30–1.72]	<0.001
Post-16 qualifications [ref yes]					
No		0.56 [0.49–0.64]	<0.001	0.71 [0.61–0.83]	<0.001
Social grade [ref ABC1]					
C2DE		0.44 [0.39–0.51]	<0.001	0.54 [0.47–0.63]	<0.001
Smoking status [ref non-smoker]					
Current smoker		0.66 [0.56–0.78]	<0.001	0.79 [0.66–0.94]	0.181
Level of alcohol consumption (AUDIT-C score)		1.17 [1.13–1.20]	<0.001	1.18 [1.14–1.21]	<0.001

*Note*: All data are weighted to match the adult population in the UK on age, social grade, region, tenure, ethnicity and working status within sex. *P*-values are adjusted for multiple comparisons using false discovery rate correction.

CI, confidence interval. OR, odds ratio. OR_adj_, odds ratio adjusted for all other variables in the table.

**Table 2:** Odds of knowing drinking guidelines are 14 units per week or fewer by survey year, sociodemographic characteristics and drinking status

		**OR [95% CI]**	***P***	**OR**_**adj**_ **[95% CI]**	***P***
Survey year [ref 2016]					
2017		0.93 [0.79–1.10]	0.574	0.93 [0.78–1.10]	0.573
2018		1.25 [1.06–1.48]	0.032	1.29 [1.09–1.53]	0.013
2019		1.22 [1.04–1.45]	0.061	1.23 [1.04–1.47]	0.056
2020		0.92 [0.79–1.09]	0.557	0.89 [0.75–1.05]	0.337
2021		0.90 [0.76–1.06]	0.382	0.88 [0.74–1.05]	0.304
2022		0.83 [0.70–0.98]	0.092	0.81 [0.68–0.96]	0.056
Age (years) [ref 18–34]					
35–64		1.59 [1.43–1.77]	<0.001	1.61 [1.44–1.80]	<0.001
≥65		1.34 [1.18–1.52]	<0.001	1.42 [1.24–1.63]	<0.001
Gender [ref male]					
Female		2.18 [2.00–2.39]	<0.001	2.24 [2.04–2.46]	<0.001
Post-16 qualifications [ref yes]					
No		0.77 [0.71–0.85]	<0.001	0.82 [0.74–0.92]	<0.001
Social grade [ref ABC1]					
C2DE		0.66 [0.60–0.72]	<0.001	0.73 [0.66–0.81]	<0.001
Smoking status [ref non-smoker]					
Current smoker		0.81 [0.72–0.91]	0.005	0.98 [0.86–1.11]	0.851
Level of alcohol consumption (AUDIT-C score)		0.98 [0.96–1.00]	0.070	1.01 [0.99–1.03]	0.330

*Note*: All data are weighted to match the adult population in the UK on age, social grade, region, tenure, ethnicity and working status within sex. *P*-values are adjusted for multiple comparisons using false discovery rate correction.

CI, confidence interval. OR, odds ratio. OR_adj_, odds ratio adjusted for all other variables in the table.

Survey year was independently associated with both awareness and knowledge of drinking guidelines. After adjusting for age, gender, education, social grade, smoking status and level of alcohol consumption, odds of being aware of the guidelines were lower among drinkers surveyed in 2019, relative to those surveyed in 2016, and significantly higher among those surveyed in 2020–2021. In contrast, odds of reporting the guideline to be 14 units per week or fewer were significantly higher among drinkers surveyed in 2018 than those surveyed in 2016 but were not significantly different from 2016 among those surveyed in other years.

Relative to younger drinkers (18–34 years), those aged 35–64 and ≥ 65 had significantly higher odds of being aware of, and accurately recalling, the guideline. There were some significant differences in trends over time: in the first year following the announcement of the revised guidelines (2016–2017), knowledge decreased among drinkers aged 35–64 compared with an increase among younger drinkers (Fig. 3A; interaction odds ratio [OR] 0.56, 95%CI 0.37–0.83). In addition, there was a decline in knowledge of guidelines over the entire study period (2016–2022) among drinkers aged ≥65 compared with little change among those aged 18–34 (Fig. 3A; interaction OR 0.51, 95%CI 0.31–0.84).

Trends in awareness and knowledge of drinking guidelines did not differ significantly by gender (Figs 2B and 3B). Across the study period, women had greater awareness and knowledge of drinking guidelines than men, although the gender disparity was substantially greater for knowledge of guidelines (224% higher odds) than for awareness (49% higher odds).

In contrast, differences by education and social grade were more pronounced for awareness than knowledge. Drinkers with no post-16 qualifications had 29% lower odds than those with post-16 qualifications of being aware of drinking guidelines but 18% lower odds of reporting the guideline to be 14 units per week or fewer. Similarly, drinkers from social grades C2DE had 46% lower odds than those from social grades ABC1 of being aware of drinking guidelines but 27% lower odds of reporting the guideline to be 14 units per week or fewer. There were no significant differences in trends over the study period by education (Figs 2C and 3C) or social grade (Figs 2D and 3D).

Across the study period, there was no significant difference in the odds of being aware of or knowing the guidelines between drinkers who did and did not smoke. However, initial changes in knowledge after the announcement of the revised guidelines varied by smoking status: between 2016 and 2017, knowledge increased among drinkers who smoked, compared with a slight decline in knowledge among non-smokers (Fig. 3E; interaction OR 2.12, 95%CI 1.36–3.32).

Relative to low-risk drinkers, increasing/higher-risk drinkers had significantly higher odds of being aware of the guidelines but similar odds of knowing the recommended weekly limit. There was a greater increase in awareness from 2016 to 2021 among drinkers with higher compared to lower levels of alcohol consumption (Fig. 2F; interaction OR 1.24, 95%CI 1.08–1.41).

Interaction results for changes between 2016 and 2019 did not differ when we excluded data collected between 2020 and 2022 from the models (Supplementary Tables 7 and 8).

**Fig. 4:**
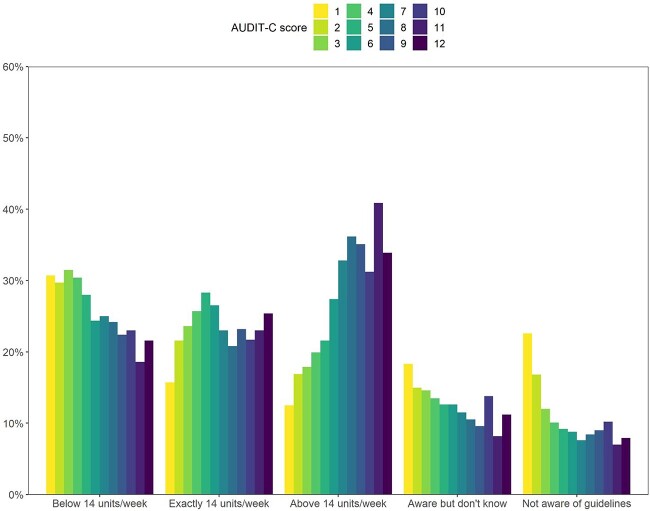
Knowledge of drinking guidelines by level of alcohol consumption. Data are from all adult drinkers in the sample who completed the AUDIT-C, 2016–2022 (*n* = 8072), weighted to match the adult population in the UK on age, social grade, region, tenure, ethnicity and working status within sex. AUDIT-C score (unweighted base *n*): 1 (*n* = 1059), 2 (*n* = 1053), 3 (*n* = 1238), 4 (*n* = 1428), 5 (*n* = 997), 6 (*n* = 731), 7 (*n* = 512), 8 (*n* = 372), 9 (*n* = 289), 10 (*n* = 245), 11 (*n* = 86), 12 (*n* = 62).

The changes in knowledge of the drinking guidelines that occurred in 2020 (Fig. 1B) coincided with a change in the profile of adult drinkers (an increased proportion of adults reporting drinking and an increased proportion of drinkers consuming at higher levels; Supplementary Table 1). We examined differences in knowledge by the level of alcohol consumption in more detail (using the five-category knowledge variable, combining data across the study period) to explore the possibility that the decline in knowledge we observed was attributable to the different population of drinkers being surveyed. Figure 4 shows that drinkers with lower levels of consumption were the least likely to know that the guideline was exactly 14 units per week and the most likely to say they were either not aware of guidelines or aware of but do not know the guidelines. Drinkers with higher levels of consumption were most likely to overestimate the recommended weekly limit.

## DISCUSSION

Between 2016 and 2022, awareness of low-risk drinking guidelines was high, with more than 8 in 10 adult drinkers in the UK saying they were aware of the guidelines. However, knowledge of the guidelines was relatively poor: only around a quarter of drinkers accurately recalled the recommended weekly limit as exactly 14 units per week and around half thought it was 14 units or fewer. Awareness of the guidelines did not increase significantly in the first few years following the announcement of the revised guidelines in January 2016: the proportion of adult drinkers in the UK who were aware of the guidelines was stable between 2016 and 2018 before falling in 2019 and increasing in 2020 to above-baseline levels. The opposite pattern was observed for knowledge of the guidelines, with the proportion of drinkers reporting the guideline to be 14 units per week or fewer increasing between 2016 and 2019, then declining. There was no improvement in inequalities in awareness and knowledge of the guidelines: disadvantaged groups remain less likely to be aware of or know the guidelines.

These results add to and extend previous evidence, which found no short-term effect of announcing the revised guidelines on awareness, but identified a modest increase in knowledge of the guidelines among men (for whom the recommended weekly limit changed) (Holmes *et al.*, 2016). Although we saw little early change in awareness, our data suggest there may have been a more gradual increase in knowledge across the whole population of drinkers over the three years following the announcement of the revised guidelines. 2020 brought about more substantial changes: increased awareness of drinking guidelines but greater uncertainty relating to the recommended weekly limit. This may have been a COVID-19 effect. The pandemic was associated with changes in the adult drinker population in the UK (Garnett *et al.*, 2021; Jackson *et al.*, 2021, 2022; Oldham *et al.*, 2021; Acuff *et al.*, 2022). The increased size of the drinker population reflects a substantial proportion of never-drinkers moving to occasional alcohol use during the pandemic; we found these people have poorer knowledge of drinking guidelines, so their inclusion in the drinker population likely contributed to (but does not fully explain) the decline in knowledge we observed. We also observed an increase in the proportion of drinkers who had post-16 qualifications, indicating higher levels of education among pandemic drinkers, which might partly account for the increase in guideline awareness. In addition, there was a shift from on-trade (i.e. licensed venues) to off-trade (i.e. shop-bought) drinking (Organization for Economic Co-operation and Development, 2021; Hardie *et al.*, 2022), which may have increased drinkers’ exposure to guidelines on product labels. The pandemic may also have increased the salience of, or drinkers’ interest in, their health and related information (including drinking guidelines). It is also possible that changes were driven in part by the change in mode of survey administration since the pandemic. Across other questions in the Alcohol Toolkit Study, there was a higher rate of ‘don’t know’ responses when participants were interviewed by telephone—although not to the same extent as observed for knowledge of drinking guidelines (e.g. the proportion of do not know responses to the first question of the AUDIT was <0.1% between 2014 and 2019 and 0.5% between 2020 and 2022).

In addition to examining overall changes in awareness and knowledge of drinking guidelines, we also explored differences across subgroups. Consistent with previous research (Office for National Statistics, 2010; Bowden *et al.*, 2014; Buykx *et al.*, 2018; Islam *et al.*, 2019), odds of being aware of and knowing the guidelines were higher among drinkers who were aged ≥35, female, more educated and more socioeconomically advantaged (social grades ABC1). Notably, drinkers who reported higher levels of alcohol consumption had significantly higher odds of being aware of the guidelines than those with lower consumption but similar odds of reporting the guideline to be 14 units per week or fewer. This paradox has been documented in other studies (Coomber *et al.*, 2017; Holmes *et al.*, 2020b), and may reflect heavier drinkers being more likely to see guidelines on product labels but rejecting or disengaging with information that threatens their freedom to drink at their usual level (Andrews, 1995; Holmes *et al.*, 2020b).

There was some evidence that trends in awareness and knowledge of guidelines differed across groups. Despite no significant changes overall in the first year following the announcement of the revised guidelines (2016–2017), there were increases in knowledge among younger drinkers (18–34 years) and those who smoked. As younger drinkers had lower levels of knowledge to begin with, this narrowed inequalities across ages—but only in the short term, as the gap in knowledge widened again post-2017. In addition, we observed a greater increase in awareness of the guidelines in 2020 among heavier drinkers. There are several possible explanations for this related to the COVID-19 pandemic as outlined above, including a change in the composition of this group (Acuff *et al.*, 2022), greater exposure to product labels due to the shift from on-trade to off-trade drinking (Organization for Economic Co-operation and  Development, 2021; Hardie *et al.*, 2022), or potentially increased information-seeking on safe alcohol use among heavier drinkers during the early stages of the pandemic.

Strengths of this study include the large, nationally representative sample, repeat cross-sectional design, and assessment of a range of relevant sociodemographic and behavioural characteristics. However, there were also limitations. First, awareness and knowledge of drinking guidelines was not assessed in every wave of the Alcohol Toolkit Study, and not in the same waves in each year (with the exception of April), which meant we had to limit our sample size in order to remove any seasonal influence on trends (there were insufficient data points to address this by simply adjusting for month of the year). However, our trends tell a broadly consistent picture despite this, with the exception of the COVID-19 effect. Secondly, other questions about the guidelines that we had included in previous papers (Holmes *et al.*, 2016, 2020c), such as where people saw the guidelines, were not included in the Alcohol Toolkit Study post-2017, so we were unable to provide updated trends here. Further research investigating where people are seeing the guidelines 6 years on from the announcement would be useful for informing interventions to improve knowledge of the guidelines. Finally, the mode of survey administration changed from face-to-face to telephone interviews in 2020 due to social distancing restrictions implemented to tackle the COVID-19 pandemic. Comparisons of the two data collection modalities generally indicate good comparability in terms of sample composition, key outcomes and associations between variables (Jackson *et al.*, 2021, 2022). However, as noted earlier, the proportion responding ‘don’t know’ to questions is higher among participants interviewed via telephone, which may account for some of the increase in uncertainty around drinking guidelines we observed post-2019.

In conclusion, 6 years since their announcement, knowledge of the revised drinking guidelines remains poor. While the majority of adult drinkers in the UK are aware of the guidelines, less than a quarter know the recommended weekly limit and only around half think it is 14 units or fewer. These data suggest passive dissemination of guidelines remains a weak implementation approach: with little improvement in knowledge since the revised guidelines were announced, increasing public awareness of low-risk drinking guidelines warrants a more proactive approach. Inequalities in awareness and knowledge of drinking guidelines have persisted over time, such that disadvantaged groups (who are at greater risk of alcohol harms) are less likely to know the guidelines. This suggests that, to date, activity to raise public awareness of the guidelines has not benefited those at highest risk. Additional interventions targeted at or tailored for disadvantaged groups may be required to address this disparity.

## AUTHOR CONTRIBUTIONS

Sarah Jackson (Conceptualization-Equal, Formal analysis-Lead, Investigation-Equal, Methodology-Equal, Visualization-Lead, Writing—original draft-Lead), Xiaotang Guo (Conceptualization-Equal, Formal analysis-Supporting, Investigation-Equal, Methodology-Equal, Writing—review and editing-Equal), John Holmes (Conceptualization-Equal, Investigation-Equal, Methodology-Equal, Writing—review and editing-Equal), Jamie Brown (Conceptualization-Equal, Data curation-Lead, Funding acquisition-Lead, Investigation-Equal, Methodology-Equal, Supervision-Lead, Writing—review and editing-Equal).

## FUNDING

Data collection was funded by Cancer Research UK (project PRCRPG-Nov21\100002) and the National Institute for Health Research (NIHR) Public Health Research (PHR) Programme (project 15/63/01). Cancer Research UK funded SJ’s salary (PRCRPG-Nov21\100002). The views expressed are those of the authors and not necessarily those of Cancer Research UK, the NIHR, or the Department of Health and Social Care.

## COMPETING INTERESTS

J.B. has received unrestricted research funding from Pfizer, who manufacture smoking cessation medications. All authors declare no financial links with the alcohol industry, tobacco companies or e-cigarette manufacturers, or their representatives.

## ETHICS APPROVAL AND CONSENT TO PARTICIPATE

Ethical approval for the STS was granted originally by the UCL Ethics Committee (ID 0498/001). The data are not collected by UCL and are anonymized when received by UCL.

## DATA AVAILABILITY

Data are available from the corresponding author on reasonable request.

## Supplementary Material

Drinking_guidelines_supplementary_material_R1_agad007
