# Supplementary material for: Trends in Public Awareness and Knowledge of Drinking Guidelines: a Representative Population Survey in England, 2016–2022
Source: Alcohol Alcohol. 2023 Feb 8;58(4):415–25. doi: 10.1093/alcalc/agad007 (PMC10331929; doi:10.1093/alcalc/agad007)
Supplement: Drinking_guidelines_supplementary_material_R1_agad007 [file drinking_guidelines_supplementary_material_r1_agad007.docx]

# Trends in public awareness and knowledge of drinking guidelines: a representative population survey in England, 2016-2022

# Supplementary material

Supplementary Table 1. Sample characteristics by survey year

Supplementary Table 2. Trends in awareness and knowledge of drinking guidelines among adult drinkers in England by survey year

Supplementary Table 3. Sample characteristics by survey year (including all available waves)

Supplementary Table 4. Trends in awareness and knowledge of drinking guidelines among adult drinkers in England by survey year (including all available waves)

Supplementary Table 5. Odds of having heard of drinking guidelines: interactions between survey year and sociodemographic characteristics and drinking status

Supplementary Table 6. Odds of knowing the drinking guidelines: interactions between survey year and sociodemographic characteristics and drinking status

Supplementary Figure 1. Trends in awareness and knowledge of drinking guidelines among adult drinkers in England by survey year (all available waves).

| **Supplementary Table 1** Sample characteristics by survey year | | | | | | | | | | |
| --- | --- | --- | --- | --- | --- | --- | --- | --- | --- | --- |
|  |  | **Total** | **2015*** | **2016** | **2017** | **2018** | **2019** | **2020** | **2021** | **2022** |
| *Total respondents, unweighted N* | | 13 659 | 3269 | 1665 | 1704 | 1724 | 1644 | 1640 | 1580 | 1587 |
|  |  |  |  |  |  |  |  |  |  |  |
| Drank any alcohol in past 6 months | |  |  |  |  |  |  |  |  |  |
|  | No | 28.7 | 31.2 | 32.6 | 33.1 | 29.5 | 31.7 | 19.5 | 24.5 | 24.4 |
|  | Yes | 70.5 | 68.8 | 67.3 | 66.8 | 67.1 | 68.1 | 80.2 | 74.0 | 73.9 |
|  | Missing | 0.8 | - | 0.1 | 0.1 | 3.5 | 0.2 | 0.3 | 1.5 | 1.6 |
|  |  |  |  |  |  |  |  |  |  |  |
| *Drinkers, unweighted N* | | 10 283 | 2115 | 1083 | 1125 | 1146 | 1098 | 1336 | 1185 | 1195 |
|  |  |  |  |  |  |  |  |  |  |  |
| Age (years) | |  |  |  |  |  |  |  |  |  |
|  | 18-34 | 27.4 | 26.1 | 28.1 | 28.8 | 28.7 | 27.3 | 27.6 | 27.7 | 26.5 |
|  | 35-54 | 50.4 | 51.9 | 51.4 | 50.7 | 49.5 | 49.6 | 49.6 | 49.1 | 50.3 |
|  | ≥65 | 22.1 | 21.9 | 20.4 | 20.6 | 21.8 | 23.1 | 22.8 | 23.2 | 23.2 |
|  | Missing | - | - | - | - | - | - | - | - | - |
|  |  |  |  |  |  |  |  |  |  |  |
| Gender | |  |  |  |  |  |  |  |  |  |
|  | Female | 48.0 | 48.3 | 46.5 | 47.6 | 47.1 | 47.1 | 49.4 | 50.0 | 48.3 |
|  | Male | 51.7 | 51.7 | 53.5 | 52.4 | 52.9 | 52.8 | 50.1 | 49.3 | 50.8 |
|  | In another way | 0.2 | - | - | - | - | 0.1 | 0.5 | 0.7 | 0.9 |
|  | Missing | - | - | - | - | - | - | - | - | - |
|  |  |  |  |  |  |  |  |  |  |  |
| Post-16 qualifications | |  |  |  |  |  |  |  |  |  |
|  | Yes | 58.9 | 53.3 | 58.0 | 58.8 | 56.2 | 62.0 | 63.9 | 60.2 | 63.3 |
|  | No | 40.3 | 46.2 | 41.7 | 40.6 | 42.8 | 38.0 | 33.4 | 38.5 | 36.2 |
|  | Missing | 0.8 | 0.5 | 0.3 | 0.6 | 0.9 | - | 2.6 | 1.3 | 0.6 |
|  |  |  |  |  |  |  |  |  |  |  |
| Social grade | |  |  |  |  |  |  |  |  |  |
|  | ABC1 | 59.7 | 60.4 | 63.9 | 62.2 | 60.5 | 62.5 | 56.6 | 56.4 | 55.5 |
|  | C2DE | 39.1 | 39.6 | 36.1 | 37.8 | 39.5 | 37.5 | 41.3 | 40.0 | 40.2 |
|  | Missing | 1.1 | - | - | - | - | - | 2.2 | 3.6 | 4.3 |
|  |  |  |  |  |  |  |  |  |  |  |
| AUDIT-C score**, mean (SD) | | 4.37 (2.56) | 4.34 (2.56) | 4.58 (2.60) | 4.42 (2.56) | 4.18 (2.55) | 4.07 (2.55) | 4.72 (2.61) | 4.21 (2.49) | 4.44 (2.50) |
|  |  |  |  |  |  |  |  |  |  |  |
| Drinking status | |  |  |  |  |  |  |  |  |  |
|  | Low risk | 58.4 | 58.9 | 56.2 | 58.1 | 61.9 | 65.3 | 51.2 | 61.3 | 54.9 |
|  | Increasing/higher risk | 40.7 | 40.9 | 43.8 | 41.6 | 38.0 | 34.2 | 47.3 | 36.3 | 42.2 |
|  | Missing | 0.9 | 0.2 | - | 0.3 | 0.1 | 0.4 | 1.5 | 2.4 | 2.9 |
|  |  |  |  |  |  |  |  |  |  |  |
| Smoking status | |  |  |  |  |  |  |  |  |  |
|  | Non-smoker | 82.1 | 79.8 | 80.6 | 83.4 | 82.5 | 84.0 | 82.6 | 82.4 | 83.4 |
|  | Current smoker | 17.7 | 20.2 | 19.4 | 16.6 | 17.5 | 16.0 | 17.2 | 17.1 | 15.2 |
|  | Missing | 0.3 | - | - | - | - | - | 0.3 | 0.5 | 1.4 |
| Note: All figures are percentages unless otherwise stated. Data are weighted to match the adult population in England on age, social grade, region, tenure, ethnicity, and working status within sex. *Data are from April for each year with the exception of 2015, where data are from November and December combined (the only available data for 2015). **AUDIT-C score: possible range 1-12. | | | | | | | | | | |

| **Supplementary Table 2** Trends in awareness and knowledge of drinking guidelines among adult drinkers in England by survey year | | | | | | | | | |
| --- | --- | --- | --- | --- | --- | --- | --- | --- | --- |
|  |  | **2015*** | **2016** | **2017** | **2018** | **2019** | **2020** | **2021** | **2022** |
| Awareness of guidelines | |  |  |  |  |  |  |  |  |
|  | *Unweighted base N* | *2115* | *1083* | *1125* | *1146* | *1098* | *1336* | *1185* | *1069* |
|  | Aware of guidelines | 86.1  [84.7-87.5] | 86.0  [84.0-88.1] | 87.0  [85.0-89.0] | 86.0  [84.0-88.0] | 81.7  [79.5-84.0] | 91.6  [90.1-93.1] | 90.5  [88.8-92.1] | 89.3  [87.5-91.1] |
|  |  |  |  |  |  |  |  |  |  |
| Knowledge of guidelines | |  |  |  |  |  |  |  |  |
|  | *Unweighted base N* | *2115* | *1083* | *1125* | *1146* | *1097* | *1329* | *1178* | *1185* |
|  | 14 units per week or fewer | 43.6  [41.5-45.6] | 52.1  [49.2-55.0] | 49.9  [47.0-52.8] | 57.4  [54.6-60.3] | 56.8  [53.9-59.7] | 49.8  [47.1-52.5] | 48.4  [45.5-51.3] | 46.5  [43.6-49.4] |
|  |  |  |  |  |  |  |  |  |  |
|  | Below 14 units per week | 23.5  [21.8-25.3] | 27.1  [24.5-29.7] | 25.9  [23.3-28.4] | 31.7  [29.0-34.3] | 31.1  [28.4-33.8] | 28.3  [25.9-30.8] | 27.2  [24.6-29.7] | 26.5  [23.9-29.0] |
|  | Exactly 14 units per week | 20.0  [18.4-21.7] | 25.0  [22.4-27.5] | 24.0  [21.5-26.5] | 25.8  [23.2-28.3] | 25.6  [23.1-28.2] | 21.2  [19.0-23.5] | 21.0  [18.6-23.3] | 19.7  [17.4-21.9] |
|  | Above 14 units per week | 32.8  [30.8-34.7] | 27.3  [24.7-29.9] | 27.2  [24.6-29.8] | 21.6  [19.2-24.0] | 19.9  [17.6-22.2] | 21.8  [19.6-24.0] | 18.4  [16.2-20.7] | 17.1  [15.0-19.3] |
|  | Aware of but don’t know drinking guidelines | 9.8  [8.6-11.0] | 6.6  [5.2-8.1] | 9.9  [8.1-11.6] | 7.0  [5.5-8.4] | 5.0  [3.8-6.3] | 19.7  [17.6-21.9] | 23.3  [20.9-25.7] | 25.2  [22.7-27.7] |
|  | Not aware of drinking guidelines | 13.9  [12.5-15.4] | 14.0  [11.9-16.0] | 13.0  [11.0-15.0] | 14.0  [12.0-16.0] | 18.3  [16.0-20.5] | 8.4  [6.9-9.9] | 9.5  [7.9-11.2] | 10.7  [8.9-12.5] |
| Note: All figures are percentages with 95% confidence intervals in parentheses unless otherwise stated. Data are weighted to match the adult population in England on age, social grade, region, tenure, ethnicity, and working status within sex. *Data are from April for each year with the exception of 2015, where data are from November and December combined (the only available data for 2015). | | | | | | | | | |

| **Supplementary Table 3** Sample characteristics by survey year (including all available waves) | | | | | | | | | | |
| --- | --- | --- | --- | --- | --- | --- | --- | --- | --- | --- |
|  |  | **Total** | **2015** | **2016** | **2017** | **2018** | **2019** | **2020** | **2021** | **2022** |
| *Drinkers, unweighted N* | | 34 265 | 2115 | 12 761 | 11 083 | 2321 | 2269 | 1336 | 1185 | 1195 |
|  |  |  |  |  |  |  |  |  |  |  |
| Age (years) | |  |  |  |  |  |  |  |  |  |
|  | 18-34 | 28.0 | 26.1 | 28.0 | 28.7 | 28.5 | 27.6 | 27.6 | 27.7 | 26.5 |
|  | 35-54 | 50.4 | 51.9 | 50.8 | 50.3 | 49.7 | 49.9 | 49.6 | 49.1 | 50.3 |
|  | ≥65 | 21.5 | 21.9 | 21.3 | 21.0 | 21.9 | 22.5 | 22.8 | 23.2 | 23.2 |
|  | Missing | - | - | - | - | - | - | - | - | - |
|  |  |  |  |  |  |  |  |  |  |  |
| Gender | |  |  |  |  |  |  |  |  |  |
|  | Female | 48.0 | 48.3 | 47.7 | 48.0 | 47.4 | 47.5 | 49.4 | 49.3 | 48.3 |
|  | Male | 51.9 | 51.7 | 52.3 | 51.9 | 52.6 | 52.3 | 50.1 | 50.0 | 50.8 |
|  | In another way | 0.1 | - | - | 0.05 | 0.02 | 0.2 | 0.5 | 0.7 | 0.9 |
|  | Missing | - | - | - | - | - | - | - | - | - |
|  |  |  |  |  |  |  |  |  |  |  |
| Post-16 qualifications | |  |  |  |  |  |  |  |  |  |
|  | Yes | 58.0 | 53.3 | 57.7 | 57.5 | 57.0 | 60.9 | 63.9 | 60.2 | 63.3 |
|  | No | 41.5 | 46.2 | 42.0 | 42.1 | 42.4 | 38.9 | 33.4 | 38.5 | 36.2 |
|  | Missing | 0.5 | 0.5 | 0.3 | 0.4 | 0.6 | 0.2 | 2.6 | 1.3 | 0.6 |
|  |  |  |  |  |  |  |  |  |  |  |
| Social grade | |  |  |  |  |  |  |  |  |  |
|  | ABC1 | 61.4 | 60.4 | 62.7 | 61.8 | 60.8 | 62.1 | 56.6 | 56.4 | 55.5 |
|  | C2DE | 38.3 | 39.6 | 37.3 | 38.2 | 39.2 | 37.9 | 41.3 | 40.0 | 40.2 |
|  | Missing | 0.3 | - | - | - | - | - | 2.2 | 3.6 | 4.3 |
|  |  |  |  |  |  |  |  |  |  |  |
| AUDIT-C score*, mean (SD) | | 4.36 (2.55) | 4.34 (2.56) | 4.41 (2.56) | 4.40 (2.56) | 4.12 (2.51) | 4.04 (2.50) | 4.72 (2.61) | 4.21 (2.49) | 4.44 (2.50) |
|  |  |  |  |  |  |  |  |  |  |  |
| Drinking status | |  |  |  |  |  |  |  |  |  |
|  | Low risk | 58.6 | 58.9 | 58.1 | 57.9 | 63.2 | 64.7 | 51.2 | 61.3 | 54.9 |
|  | Increasing/higher risk | 40.9 | 40.9 | 41.6 | 41.8 | 36.6 | 35.0 | 47.3 | 36.3 | 42.2 |
|  | Missing | 0.5 | 0.2 | 0.3 | 0.3 | 0.2 | 0.3 | 1.5 | 2.4 | 2.9 |
|  |  |  |  |  |  |  |  |  |  |  |
| Smoking status | |  |  |  |  |  |  |  |  |  |
|  | Non-smoker | 82.4 | 79.8 | 81.5 | 82.9 | 83.4 | 85.5 | 82.6 | 82.4 | 83.4 |
|  | Current smoker | 17.5 | 20.2 | 18.5 | 17.0 | 16.6 | 14.5 | 17.2 | 17.1 | 15.2 |
|  | Missing | 0.1 | - | - | 0.04 | - | - | 0.3 | 0.5 | 1.4 |
| Note: All figures are percentages unless otherwise stated. Data are weighted to match the adult population in England on age, social grade, region, tenure, ethnicity, and working status within sex. *AUDIT-C score: possible range 1-12. | | | | | | | | | | |

| **Supplementary Table 4** Trends in awareness and knowledge of drinking guidelines among adult drinkers in England by survey year (including all available waves) | | | | | | | | | |
| --- | --- | --- | --- | --- | --- | --- | --- | --- | --- |
|  |  | **2015** | **2016** | **2017** | **2018** | **2019** | **2020** | **2021** | **2022** |
| Awareness of guidelines | |  |  |  |  |  |  |  |  |
|  | *Unweighted base N* | *2115* | *12 761* | *11 083* | *2321* | *2269* | *1336* | *1185* | *1069* |
|  | Aware of guidelines | 86.1  [84.7-87.5] | 87.9  [87.3-88.5] | 86.3  [85.6-86.9] | 86.1  [84.7-87.5] | 82.8  [81.2-84.3] | 91.6  [90.1-93.1] | 90.5  [88.8-92.1] | 89.3  [87.5-91.1] |
|  |  |  |  |  |  |  |  |  |  |
| Knowledge of guidelines | |  |  |  |  |  |  |  |  |
|  | *Unweighted base N* | *2115* | *12 758* | *11 080* | *2321* | *2265* | *1329* | *1178* | *1185* |
|  | 14 units per week or fewer | 43.6  [41.5-45.6] | 52.0  [51.1-52.8] | 50.9  [49.9-51.8] | 56.1  [54.1-58.1] | 55.8  [53.7-57.8] | 49.8  [47.1-52.5] | 48.4  [45.5-51.3] | 46.5  [43.6-49.4] |
|  |  |  |  |  |  |  |  |  |  |
|  | Below 14 units per week | 23.5  [21.8-25.3] | 26.6  [25.9-27.4] | 26.5  [25.7-27.3] | 30.9  [29.0-32.7] | 29.8  [28.0-31.7] | 28.3  [25.9-30.8] | 27.2  [24.6-29.7] | 26.5  [23.9-29.0] |
|  | Exactly 14 units per week | 20.0  [18.4-21.7] | 25.3  [24.6-26.1] | 24.4  [23.6-25.2] | 25.3  [23.5-27.0] | 25.8  [24.0-27.6] | 21.2  [19.0-23.5] | 21.0  [18.6-23.3] | 19.7  [17.4-21.9] |
|  | Above 14 units per week | 32.8  [30.8-34.7] | 27.3  [26.5-28.0] | 27.9  [27.1-28.8] | 22.0  [20.4-23.7] | 20.5  [18.8-22.2] | 21.8  [19.6-24.0] | 18.4  [16.2-20.7] | 17.1  [15.0-19.3] |
|  | Aware of but don’t know drinking guidelines | 9.8  [8.6-11.0] | 8.7  [8.2-9.2] | 7.5  [7.0-7.9] | 7.9  [6.8-9.0] | 6.4  [5.4-7.4] | 19.7  [17.6-21.9] | 23.3  [20.9-25.7] | 25.2  [22.7-27.7] |
|  | Not aware of drinking guidelines | 13.9  [12.5-15.4] | 12.1  [11.5-12.7] | 13.7  [13.1-14.4] | 13.9  [12.5-15.3] | 17.3  [15.7-18.8] | 8.4  [6.9-9.9] | 9.5  [7.9-11.2] | 12.1  [11.5-12.7] |
| Note: All figures are percentages with 95% confidence intervals in parentheses unless otherwise stated. Data are weighted to match the adult population in England on age, social grade, region, tenure, ethnicity, and working status within sex. | | | | | | | | | |

| **Supplementary Table 5** Odds of being aware of drinking guidelines: interactions between survey year and sociodemographic characteristics and drinking status | | | | | | | | | |  |
| --- | --- | --- | --- | --- | --- | --- | --- | --- | --- | --- |
|  |  | **Age 35-64 [ref 18-34]** | |  | **Age 65+ [ref 18-34]** | |  | **Female [ref male]** | | |
|  |  | **OR_adj_ [95% CI]** | ***p*** |  | **OR_adj_ [95% CI]** | ***p*** |  | **OR_adj_ [95% CI]** | ***p*** | |
| Survey year [ref 2016] | |  |  |  |  |  |  |  |  | |
|  | 2017 | 0.77 [0.43-1.37] | 0.573 |  | 0.57 [0.29-1.11] | 0.220 |  | 0.86 [0.52-1.44] | 0.747 | |
|  | 2018 | 0.76 [0.44-1.33] | 0.573 |  | 0.75 [0.39-1.44] | 0.574 |  | 0.70 [0.43-1.15] | 0.321 | |
|  | 2019 | 0.71 [0.42-1.22] | 0.393 |  | 1.11 [0.58-2.12] | 0.851 |  | 0.85 [0.53-1.36] | 0.683 | |
|  | 2020 | 1.40 [0.74-2.66] | 0.519 |  | 0.89 [0.43-1.81] | 0.851 |  | 0.85 [0.49-1.49] | 0.747 | |
|  | 2021 | 1.88 [0.97-3.62] | 0.159 |  | 0.95 [0.47-1.89] | 0.957 |  | 1.25 [0.71-2.21] | 0.625 | |
|  | 2022 | 0.85 [0.45-1.61] | 0.798 |  | 0.47 [0.23-0.93] | 0.091 |  | 0.85 [0.49-1.45] | 0.739 | |
|  |  | **No post-16 qualifications [ref yes]** | |  | **Social grade C2DE [ref ABC1]** | |  | **Level of alcohol consumption (AUDIT-C score)** | | |
|  |  | **OR_adj_ [95% CI]** | ***p*** |  | **OR_adj_ [95% CI]** | ***p*** |  | **OR_adj_ [95% CI]** | ***p*** | |
| Survey year [ref 2016] | |  |  |  |  |  |  |  |  | |
|  | 2017 | 1.32 [0.80-2.18] | 0.492 |  | 1.68 [1.01-2.77] | 0.125 |  | 0.97 [0.88-1.07] | 0.719 | |
|  | 2018 | 0.97 [0.60-1.59] | 0.976 |  | 0.89 [0.54-1.45] | 0.806 |  | 1.11 [1.00-1.23] | 0.147 | |
|  | 2019 | 0.98 [0.61-1.57] | 0.976 |  | 1.01 [0.63-1.62] | 0.976 |  | 1.01 [0.92-1.11] | 0.927 | |
|  | 2020 | 1.10 [0.63-1.92] | 0.851 |  | 0.90 [0.51-1.59] | 0.851 |  | 1.15 [1.02-1.29] | 0.080 | |
|  | 2021 | 1.23 [0.71-2.13] | 0.645 |  | 0.91 [0.52-1.58] | 0.851 |  | **1.24 [1.08-1.41]** | **0.009** | |
|  | 2022 | 1.08 [0.63-1.84] | 0.882 |  | 1.58 [0.92-2.69] | 0.220 |  | 1.09 [0.97-1.22] | 0.277 | |
|  |  | **Current smoker [ref no]** | |  |  | |  |  | | |
|  |  | **OR_adj_ [95% CI]** | ***p*** |  |  |  |  |  |  | |
| Survey year [ref 2016] | |  |  |  |  |  |  |  |  | |
|  | 2017 | 1.42 [0.77-2.65] | 0.466 |  |  |  |  |  |  | |
|  | 2018 | 0.89 [0.50-1.60] | 0.851 |  |  |  |  |  |  | |
|  | 2019 | 1.02 [0.58-1.80] | 0.976 |  |  |  |  |  |  | |
|  | 2020 | 0.89 [0.47-1.69] | 0.851 |  |  |  |  |  |  | |
|  | 2021 | 1.21 [0.62-2.35] | 0.747 |  |  |  |  |  |  | |
|  | 2022 | 1.24 [0.64-2.41] | 0.719 |  |  |  |  |  |  | |
| Note: All data are weighted to match the adult population in England on age, social grade, region, tenure, ethnicity, and working status within sex. P values are adjusted for multiple comparisons using false discovery rate correction (applied excluding sensitivity analyses reported in Supplementary Tables 7 and 8). Bold text indicates significant interactions. CI, confidence interval. OR_adj_, odds ratio adjusted for age, gender, education, social grade, level of alcohol consumption, and smoking status. | | | | | | | | | |  |

| **Supplementary Table 6** Odds of knowing the drinking guidelines: interactions between survey year and sociodemographic characteristics and drinking status | | | | | | | | | |  |
| --- | --- | --- | --- | --- | --- | --- | --- | --- | --- | --- |
|  |  | **Age 35-64 [ref 18-34]** | |  | **Age 65+ [ref 18-34]** | |  | **Female [ref male]** | | |
|  |  | **OR_adj_ [95% CI]** | ***p*** |  | **OR_adj_ [95% CI]** | ***p*** |  | **OR_adj_ [95% CI]** | ***p*** | |
| Survey year [ref 2016] | |  |  |  |  |  |  |  |  | |
|  | 2017 | **0.56 [0.37-0.83]** | **0.017** |  | 0.58 [0.36-0.96] | 0.094 |  | 1.31 [0.93-1.84] | 0.274 | |
|  | 2018 | 0.93 [0.62-1.39] | 0.851 |  | 0.66 [0.41-1.08] | 0.223 |  | 1.06 [0.75-1.50] | 0.851 | |
|  | 2019 | 1.20 [0.80-1.80] | 0.574 |  | 0.98 [0.60-1.61] | 0.976 |  | 1.05 [0.75-1.49] | 0.859 | |
|  | 2020 | 0.77 [0.52-1.14] | 0.370 |  | 0.64 [0.40-1.03] | 0.168 |  | 1.00 [0.72-1.40] | 1.000 | |
|  | 2021 | 1.02 [0.68-1.54] | 0.976 |  | 0.69 [0.42-1.13] | 0.291 |  | 0.72 [0.51-1.01] | 0.149 | |
|  | 2022 | 0.68 [0.45-1.03] | 0.168 |  | **0.51 [0.31-0.84]** | **0.035** |  | 1.07 [0.75-1.51] | 0.851 | |
|  |  | **No post-16 qualifications [ref yes]** | |  | **Social grade C2DE [ref ABC1]** | |  | **Level of alcohol consumption (AUDIT-C score)** | | |
|  |  | **OR_adj_ [95% CI]** | ***p*** |  | **OR_adj_ [95% CI]** | ***p*** |  | **OR_adj_ [95% CI]** | ***p*** | |
| Survey year [ref 2016] | |  |  |  |  |  |  |  |  | |
|  | 2017 | 0.98 [0.70-1.39] | 0.976 |  | 1.38 [0.97-1.97] | 0.177 |  | 0.99 [0.93-1.06] | 0.859 | |
|  | 2018 | 0.99 [0.70-1.40] | 0.976 |  | 0.85 [0.60-1.21] | 0.573 |  | 1.03 [0.97-1.10] | 0.557 | |
|  | 2019 | 0.88 [0.62-1.25] | 0.676 |  | 1.15 [0.80-1.64] | 0.643 |  | 1.04 [0.97-1.11] | 0.446 | |
|  | 2020 | 1.06 [0.75-1.50] | 0.851 |  | 1.17 [0.83-1.65] | 0.573 |  | 1.03 [0.97-1.10] | 0.574 | |
|  | 2021 | 1.40 [0.98-1.99] | 0.159 |  | 1.01 [0.71-1.44] | 0.976 |  | 1.08 [1.01-1.15] | 0.093 | |
|  | 2022 | 1.16 [0.81-1.65] | 0.625 |  | 1.38 [0.97-1.98] | 0.177 |  | 1.04 [0.97-1.11] | 0.437 | |
|  |  | **Current smoker [ref no]** | |  |  | |  |  | | |
|  |  | **OR_adj_ [95% CI]** | ***p*** |  |  |  |  |  |  | |
| Survey year [ref 2016] | |  |  |  |  |  |  |  |  | |
|  | 2017 | **2.12 [1.36-3.32]** | **0.005** |  |  |  |  |  |  | |
|  | 2018 | 1.39 [0.89-2.15] | 0.294 |  |  |  |  |  |  | |
|  | 2019 | 0.99 [0.63-1.56] | 0.977 |  |  |  |  |  |  | |
|  | 2020 | 1.39 [0.90-2.15] | 0.291 |  |  |  |  |  |  | |
|  | 2021 | 0.69 [0.44-1.09] | 0.246 |  |  |  |  |  |  | |
|  | 2022 | 1.70 [1.07-2.69] | 0.078 |  |  |  |  |  |  | |
| Note: All data are weighted to match the adult population in England on age, social grade, region, tenure, ethnicity, and working status within sex. P values are adjusted for multiple comparisons using false discovery rate correction (applied excluding sensitivity analyses reported in Supplementary Tables 7 and 8). Bold text indicates significant interactions. CI, confidence interval. OR_adj_, odds ratio adjusted for age, gender, education, social grade, drinking status, and smoking status. | | | | | | | | | |  |

| **Supplementary Table 7** Odds of being aware of drinking guidelines: interactions between survey year and sociodemographic characteristics and drinking status, 2016-2019 only | | | | | | | | | |  |
| --- | --- | --- | --- | --- | --- | --- | --- | --- | --- | --- |
|  |  | **Age 35-64 [ref 18-34]** | |  | **Age 65+ [ref 18-34]** | |  | **Female [ref male]** | | |
|  |  | **OR_adj_ [95% CI]** | ***p*** |  | **OR_adj_ [95% CI]** | ***p*** |  | **OR_adj_ [95% CI]** | ***p*** | |
| Survey year [ref 2016] | |  |  |  |  |  |  |  |  | |
|  | 2017 | 0.77 [0.43-1.37] | 0.570 |  | 0.57 [0.29-1.12] | 0.202 |  | 0.87 [0.52-1.44] | 0.753 | |
|  | 2018 | 0.78 [0.45-1.35] | 0.570 |  | 0.76 [0.40-1.46] | 0.595 |  | 0.72 [0.44-1.18] | 0.873 | |
|  | 2019 | 0.72 [0.42-1.22] | 0.404 |  | 1.12 [0.59-2.14] | 0.873 |  | 0.85 [0.53-1.37] | 0.944 | |
|  |  | **No post-16 qualifications [ref yes]** | |  | **Social grade C2DE [ref ABC1]** | |  | **Level of alcohol consumption (AUDIT-C score)** | | |
|  |  | **OR_adj_ [95% CI]** | ***p*** |  | **OR_adj_ [95% CI]** | ***p*** |  | **OR_adj_ [95% CI]** | ***p*** | |
| Survey year [ref 2016] | |  |  |  |  |  |  |  |  | |
|  | 2017 | 1.30 [0.79-2.14] | 0.532 |  | 1.68 [1.02-2.76] | 0.109 |  | 0.97 [0.88-1.07] | 0.723 | |
|  | 2018 | 0.97 [0.59-1.57] | 0.968 |  | 0.90 [0.55-1.47] | 0.852 |  | 1.11 [1.00-1.23] | 0.124 | |
|  | 2019 | 0.98 [0.62-1.57] | 0.994 |  | 1.00 [0.63-1.61] | 0.994 |  | 1.01 [0.92-1.11] | 0.944 | |
|  |  | **Current smoker [ref no]** | |  |  | |  |  | | |
|  |  | **OR_adj_ [95% CI]** | ***p*** |  |  |  |  |  |  | |
| Survey year [ref 2016] | |  |  |  |  |  |  |  |  | |
|  | 2017 | 1.39 [0.75-2.57] | 0.527 |  |  |  |  |  |  | |
|  | 2018 | 0.91 [0.51-1.62] | 0.875 |  |  |  |  |  |  | |
|  | 2019 | 1.02 [0.58-1.79] | 0.994 |  |  |  |  |  |  | |
| Note: All data are weighted to match the adult population in England on age, social grade, region, tenure, ethnicity, and working status within sex. P values are adjusted for multiple comparisons using false discovery rate correction (applied excluding interaction results reported in Supplementary Tables 5 and 6). Bold text indicates significant interactions. CI, confidence interval. OR_adj_, odds ratio adjusted for age, gender, education, social grade, level of alcohol consumption, and smoking status. | | | | | | | | | |  |

| **Supplementary Table 8** Odds of knowing the drinking guidelines: interactions between survey year and sociodemographic characteristics and drinking status, 2016-2019 only | | | | | | | | | |  |
| --- | --- | --- | --- | --- | --- | --- | --- | --- | --- | --- |
|  |  | **Age 35-64 [ref 18-34]** | |  | **Age 65+ [ref 18-34]** | |  | **Female [ref male]** | | |
|  |  | **OR_adj_ [95% CI]** | ***p*** |  | **OR_adj_ [95% CI]** | ***p*** |  | **OR_adj_ [95% CI]** | ***p*** | |
| Survey year [ref 2016] | |  |  |  |  |  |  |  |  | |
|  | 2017 | **0.55 [0.37-0.82]** | **0.010** |  | 0.58 [0.35-0.95] | 0.083 |  | 1.31 [0.93-1.85] | 0.240 | |
|  | 2018 | 0.92 [0.61-1.38] | 0.856 |  | 0.66 [0.40-1.07] | 0.188 |  | 1.07 [0.76-1.51] | 0.863 | |
|  | 2019 | 1.20 [0.80-1.81] | 0.571 |  | 0.98 [0.60-1.61] | 0.994 |  | 1.06 [0.75-1.49] | 0.878 | |
|  |  | **No post-16 qualifications [ref yes]** | |  | **Social grade C2DE [ref ABC1]** | |  | **Level of alcohol consumption (AUDIT-C score)** | | |
|  |  | **OR_adj_ [95% CI]** | ***p*** |  | **OR_adj_ [95% CI]** | ***p*** |  | **OR_adj_ [95% CI]** | ***p*** | |
| Survey year [ref 2016] | |  |  |  |  |  |  |  |  | |
|  | 2017 | 0.98 [0.70-1.39] | 0.994 |  | 1.39 [0.98-1.99] | 0.152 |  | 0.99 [0.93-1.06] | 0.888 | |
|  | 2018 | 0.99 [0.70-1.40] | 0.994 |  | 0.85 [0.60-1.21] | 0.570 |  | 1.03 [0.97-1.10] | 0.570 | |
|  | 2019 | 0.88 [0.62-1.25] | 0.690 |  | 1.14 [0.80-1.63] | 0.671 |  | 1.04 [0.97-1.11] | 0.474 | |
|  |  | **Current smoker [ref no]** | |  |  | |  |  | | |
|  |  | **OR_adj_ [95% CI]** | ***p*** |  |  |  |  |  |  | |
| Survey year [ref 2016] | |  |  |  |  |  |  |  |  | |
|  | 2017 | **2.16 [1.37-3.38]** | **0.004** |  |  |  |  |  |  | |
|  | 2018 | 1.42 [0.91-2.21] | 0.233 |  |  |  |  |  |  | |
|  | 2019 | 1.00 [0.64-1.58] | 0.994 |  |  |  |  |  |  | |
| Note: All data are weighted to match the adult population in England on age, social grade, region, tenure, ethnicity, and working status within sex. P values are adjusted for multiple comparisons using false discovery rate correction (applied excluding interaction results reported in Supplementary Tables 5 and 6). Bold text indicates significant interactions. CI, confidence interval. OR_adj_, odds ratio adjusted for age, gender, education, social grade, drinking status, and smoking status. | | | | | | | | | |  |


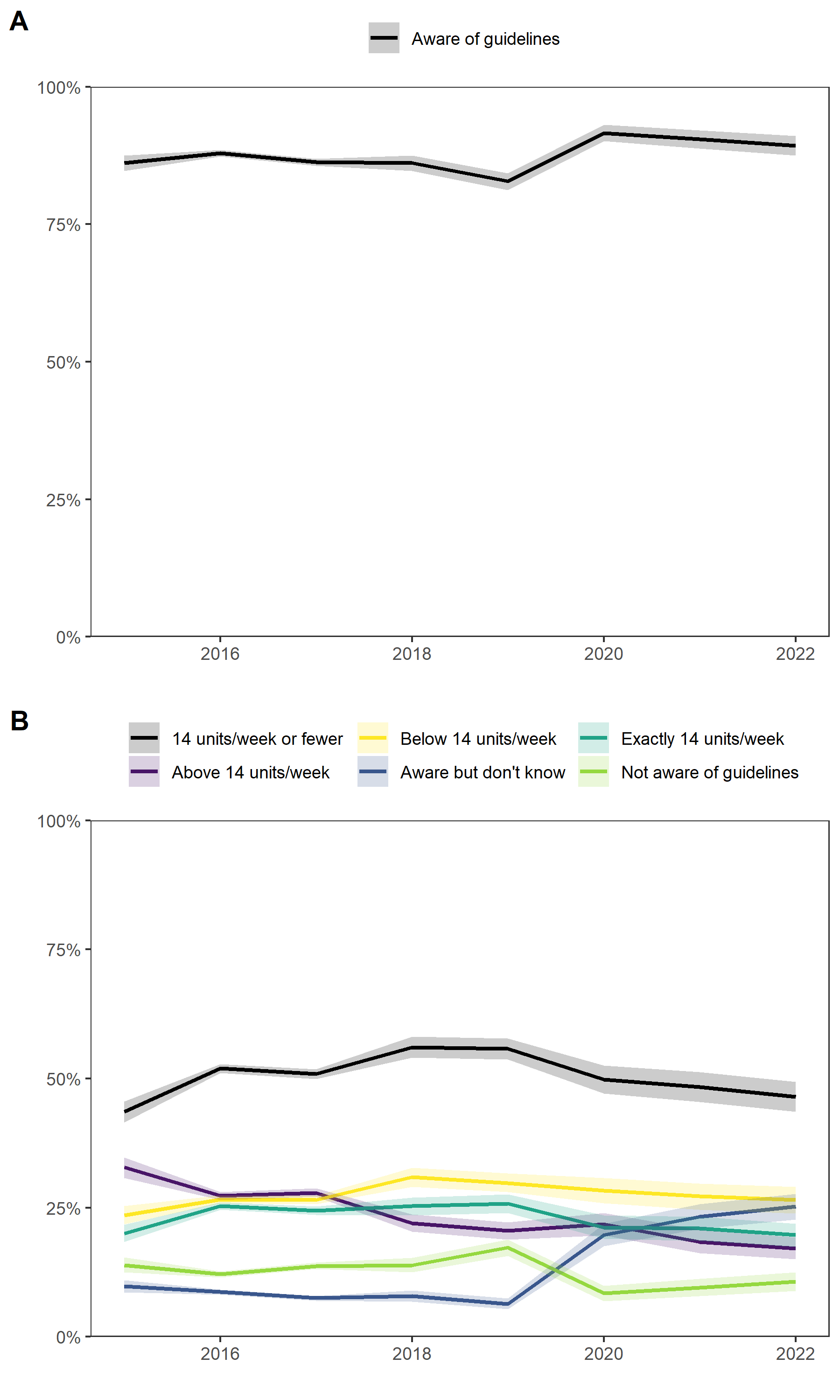


**Supplementary Figure 1 Trends in awareness and knowledge of drinking guidelines among adult drinkers in England by survey year (all available waves)**. Lines represent the weighted prevalence of (A) awareness and (B) knowledge of drinking guidelines. Shaded bands indicate the 95% confidence interval. Note: In panel B, the line for *14 units/week or fewer* combines respondents who report the guideline to be exactly or below 14 units per week.
